# Effects of Ninjin'yoeito on Human *CYP3A* and Mouse *CYP3A* Activity

**DOI:** 10.1155/2023/8657478

**Published:** 2023-08-02

**Authors:** Marisa Kaneda, Manami Oyama, Takashi Yoshimi, Seiwa Michihara, Li-Kun Han, Nina Fujita, Ryuji Takahashi

**Affiliations:** Kampo Research Laboratories, Kracie Pharma, Ltd., 3-1 Kanebo-machi, Takaoka-shi, Toyama 933-0856, Japan

## Abstract

Ninjin'yoeito (NYT) is widely used clinically for the management of patients with frailty and other multiple symptoms. NYT is often administered with other drugs; however, little information is available on its drug interactions. Previous studies using human liver microsomes have reported that constituents of NYT either inhibit (schisandra fruit, cinnamon bark, glycyrrhiza, and poria sclerotium) or induce (schisandra fruit and glycyrrhiza) CYP3A4 expression. Herein, we conducted in vitro and in vivo studies targeting human CYP3A and mouse CYP3A to elucidate the effects of NYT coadministration with other drugs on hepatic drug metabolism. In an inhibition study using human liver microsomes, NYT showed concentration-dependent reversible inhibition and time-dependent inhibition. Furthermore, in an induction study using frozen human hepatocytes, the addition of 0.01–0.1 mg/mL NYT resulted in a concentration-dependent increase in CYP3A gene expression. Contrarily, no significant changes in CYP3A substrate blood concentrations were observed between untreated mice and mice that received either a single dose of NYT or repeated doses for 15 days. These results demonstrate that NYT has inhibitory and inductive effects on hepatic CYP3A in vitro, but orally administered NYT does not affect drug metabolism mediated by hepatic CYP3A in vivo in the mouse model. Although there is a little information about drug interactions of NYT, this study provides new evidence for that.

## 1. Introduction

In recent years, the demand for Kampo medicines has increased along with progress in pharmacological research and an increase in the number of patients with chronic diseases [1]. Ninjin'yoeito (NYT) is a traditional Kampo medicine consisting of 12 types of crude drugs (Table 1).

In Japan, NYT is prescribed for patients with a weakened constitution following disease recovery, fatigue and malaise, anorexia, perspiration during sleep, cold limbs, and anemia. Recent clinical studies in older adults have reported that NYT alleviates anorexia, loss of grip strength, and postoperative general conditions [2–5]. Thus, NYT has been postulated as a therapeutic option for frailty symptoms associated with physical weakness [6]. In an aging society, numerous patients suffer from multiple diseases. NYT is effective in treating diseases and various symptoms associated with aging and is often used in combination with Western drugs to alleviate these conditions. Generally, drug interactions should be considered when multiple drugs are administered together. However, little information is available on the drug interactions of Kampo medicines, including NYT.

During treatment with multiple agents, it is important to understand the characteristics of each drug and recognize the occurrence of adverse events [7]. However, adverse events and decreased efficacy are particularly difficult to predict based on the pharmacokinetic mechanisms among drug interactions. Orally administered drugs are mainly absorbed in the gastrointestinal tract, transported to the liver where they undergo first-pass metabolism, and then enter systemic circulation. Various transporters and metabolic enzymes are involved in this complex process with effects on drug clearance. Among these enzymes, cytochrome P450 (CYP) is one of the most important, especially in drug interactions [8]. CYPs are known to be involved in approximately 80% of drug metabolism reactions, with CYP3A being involved in the metabolism of approximately 50% or more of drugs currently in clinical use. Since there is little information on drug interactions of NYT, we aimed to provide new evidence for that. In this study, we investigated the possible effect of interaction between NYT and other drugs on human CYP3A and mouse CYP3A in vitro and in vivo.

## 2. Materials and Methods

### 2.1. Reagents

Human liver microsomes (XTreme200 Human Liver Microsomes Pool of 200; 100 males and 100 females; Sekisui Medical Co., Ltd., Tokyo, Japan) were used in the in vitro inhibition study. Testosterone (TES; Sigma-Aldrich, St. Louis, MO, USA), 6*β*-hydroxytestosterone (OHTES; Sigma-Aldrich), and 6*β*-hydroxytestosterone-d7 (OHTES- d7; CORNING, Corning, NY, USA) were used as the substrate, metabolite (calibration standard), and internal standard, respectively. Grapefruit juice (GFJ) extract was obtained by freeze-drying commercial GFJ (Tropicana, Lot No. +KKH/2E058C; Kirin Beverage Company, Limited, Kyoto, Japan). After weighing GFJ and NYT, distilled water was added to reach 10 mg/mL and 20 mg/mL, respectively. They were then centrifuged at 10,000 × g for 1 min, and the supernatant was treated as the test substance stock solution. The stock solution and diluted solution were prepared as needed. Frozen human hepatocytes (Thermo Fisher Scientific, Waltham, MA, USA; Lot No. HU8300) were used in the in vitro induction study. For the cell culture, cryopreserved hepatocyte recovery medium (CHRM) (A1217601; Thermo Fisher Scientific) containing primary hepatocyte thawing and plating supplements (CM3000; Thermo Fisher Scientific) or primary hepatocyte maintenance supplements (CM4000; Thermo Fisher Scientific) was used as the seeding media or induction media, respectively. Rifampicin was used as a positive control. In vivo, triazolam (TRZ; FUJIFILM Wako, Osaka, Japan) was used as the substrate and ketoconazole (FUJIFILM Wako) was used as the positive control (known inhibitor). Alprazolam (ALP; FUJIFILM Wako) was used as the internal standard on HPLC.

### 2.2. Plant Materials

NYT extract powder (Lot No. 15112017) was manufactured by the GMP Pharmaceutical Factory of Kracie Pharma, Ltd. (Qingdao, China). The quality of each plant material was identified by external morphology and authenticated by marker compounds of the plant specimens (e.g., glycyrrhizic acid, paeoniflorin, and hesperidin) according to the Japanese Pharmacopoeia and standards of Kracie Pharma, Ltd. After preparation, the profile of the NYT extract was the same as those reported previously when examined using the analytical method and 3D high-performance liquid chromatography (HPLC) [9].

### 2.3. Measurement of CYP3A Activity In Vitro

The in vitro study on the CYP3A inhibitory and inductive activities was outsourced to Kamakura Techno-Science, Inc. (Kanagawa, Japan). To assess reversible inhibition, a reaction mixture containing 100 mmol/L phosphate buffer (pH 7.4; Corning), 0.03 mg/mL microsomal protein, and 88 *µ*mol/L substrate (TES) was prepared. Diluted GFJ solutions (0, 0.05, 0.1, 0.5, 1, and 5 mg/mL in distilled water) or diluted NYT solutions (0, 0.01, 0.1, 1, and 10 mg/mL in distilled water) were added to the reaction mixture and prewarmed for 5 min at 37°C. The metabolic reaction was allowed to proceed by adding 20 mmol/L aqueous nicotinamide adenine dinucleotide phosphate (NADPH) solution and incubating at 37°C for 10 min, and the internal standard solution (100 nmol/L OHTES-d7) was added to terminate the reaction. The mixture was centrifuged at 1,900 × g and 4°C for 5 min, the supernatant was diluted 2.5-fold with distilled water, and the resulting solution was used for measurements as the metabolic reaction sample. On the other hand, the background sample (BG) was the one to which 20 mmol/L NADPH solution was added after the reaction was stopped without incubation. To assess time-dependent inhibition (TDI), a reaction mixture containing 100 mmol/L phosphate buffer (pH 7.4; Corning), 0.03 mg/mL microsomal protein, and 1 *µ*mol/L NADPH was prepared and prewarmed for 5 min at 37°C. Diluted GFJ solutions (0, 0.05, 0.1, 0.5, 1, and 5 mg/mL in distilled water) or diluted NYT solutions (0, 0.01, 0.1, 1, and 10 mg/mL in distilled water) were added to the reaction mixture, and the mixture was preincubated for 30 min at 37°C. Subsequently, 1,760 *µ*mol/L TES was added, and the metabolic reaction was allowed to proceed by incubating at 37°C for 10 min prior to adding the internal standard solution (100 nmol/L OHTES-d7) to terminate the reaction. The mixture was centrifuged at 1,900 × g and 4°C for 5 min, the supernatant was diluted 2.5-fold with distilled water, and the resulting solution was used for measurements as the metabolic reaction sample.

### 2.4. Measurement of Metabolic Activity and the Half Maximal Inhibitory Concentration (IC_50_)

Standard samples for the calibration curve and metabolic reaction samples were quantified using liquid chromatography/mass spectrometry (HPLC; Shimazu Corporation, Kyoto, Japan, MS/MS; AB Sciex Pte, Tokyo, Japan) (Table 2). Standard solutions for the calibration curve were prepared by diluting a 400 *µ*mol/L OHTES stock solution (50% v/v acetonitrile) with 25% v/v acetonitrile. The metabolic activity was calculated using the following formula:

(1) $$\begin{array}{c} E=\frac{Bt-BG}{Nt-BG}\times 100\text{.} \end{array}$$

E: metabolic activity at inhibitor concentration *n* (%), Bt: metabolite concentration after incubation at inhibitor concentration *n*, Nt: metabolite concentration after incubation at the time of nonaddition of the inhibitor, and BG: metabolite concentration (mean) in the background sample.

Metabolite concentrations below the lower limit of quantitation were treated as zero. The IC_50_ value was calculated using the metabolic activity and concentration of the inhibitor and expressed to three significant figures. Statistical analysis software Exsus (version 8.1.0, CAC Exicare; EPS Corporation. Tokyo, Japan) linked to the SAS system (version 9.4) was used to calculate the IC_50_ values.

### 2.5. Quantification of mRNA

Frozen human hepatocytes were rapidly thawed in a water bath at 37°C and seeded in 24-well plates at 3.5 × 10^6^ cells/well with a cell-seeding medium. After culturing for 24 h, the medium was changed to the NYT-added medium. After incubation for 72 h, while changing the medium every 24 h, total RNA was collected. cDNA was prepared using the RNeasy® mini kit (QIAGEN, Hilden, Germany), QIAshredder™ spin column (QIAGEN), and SuperScript® VILO cDNA synthesis kit (Thermo Fisher Scientific) according to the manufacturer's protocols. TaqMan™ master mix (Thermo Fisher Scientific) was used for real-time PCR (QuantStudioTM 12K Flex; Thermo Fisher Scientific). The value was corrected using the house-keeping gene GAPDH, and this was utilized as the CYP3A4 gene expression level.

### 2.6. Animal Preparation and Experimental Protocol

For the animal experiments, 6–7-week-old male ddY mice were purchased from Japan SLC, Inc. (Shizuoka, Japan). The animals were reared at a temperature of 23 ± 2°C and humidity of 55 ± 10% under a 12-h light-dark cycle (lights on from 8:00 to 20:00). They were allowed to feed freely on food (CE-2; CLEA Japan, Inc., Tokyo, Japan) and water. All experiments were performed with an effort to minimize suffering and the number of animals used. The animal experimental protocol was reviewed and approved by the Animal Experiment Care Committee of Kracie Pharma, Ltd. and carried out in accordance with animal experimentation rules stipulated by the institute as well as the “Basic Guidelines for Implementation of Animal Experiments at Institutions under the Jurisdiction of the Ministry of Health, Labour, and Welfare” (internal approval nos. 210045 and 210046).

### 2.7. Drug Treatment

In the single-dose test, after acclimatization for one week, the animals were divided into control, ketoconazole, NYT 1 h preadministration, and NYT 2 h preadministration groups (*n* = 26 per group). Mice were made to fast for 16 h from the evening of the day before necropsy. TRZ and ketoconazole were dissolved in 10% PEG-400/saline. NYT suspension (560 mg/kg body weight; B.W.) was orally administered 1 h (NYT 1 h preadministration group) or 2 h (NYT 2 h preadministration group) before TRZ administration. Similarly, distilled water was orally administrated to the control and ketoconazole groups. In addition, ketoconazole suspension (100 mg/kg B.W.) or the solvent was administered intraperitoneally to the ketoconazole group or the other groups, respectively. One hour after intraperitoneal administration, TRZ (0.3 mg/kg B.W.) was intraperitoneally administered, and blood was collected from the abdominal vena cava under deep isoflurane anesthesia at 0, 15-, 30-, 45-, and 60-minute time points by using a heparin-coated syringe. Blood samples were centrifuged at 5,000 × g and 4°C for 10 min to obtain plasma samples. In the repeated-dose test, after acclimatization for one week, the animals were divided into control, ketoconazole, and NYT groups (*n* = 22–24 per group). Distilled water was administered to the control and ketoconazole groups, and NYT suspension (1,700 mg/kg B.W./day) was administered to the NYT group for 15 days until the day before the test. On the day after the last administration, ketoconazole suspension (100 mg/kg B.W.) was intraperitoneally administered to the ketoconazole group and 10% PEG-400/saline to the other groups 1 h before TRZ administration. Thereafter, the same procedures as those outlined in the single-dose test were performed.

### 2.8. HPLC Analysis of Blood TRZ Concentrations

Blood TRZ concentrations were measured using a previously published method [10] with slight modifications. ALP, which has a similar structure to TRZ, was used as the internal standard. Thirty microliters of 1,000 ng/mL ALP and 330 *μ*L of 2-propanol were added to 300 *μ*L of plasma, which was allowed to stand for 10 min at 4°C and then centrifuged at 4°C, 10,000 × g for 5 min. To the supernatant, 450 *µ*L of borate buffer (50 mM, pH 11.0) and 1 mL of chloroform were added and then shaken for 10 min before separating the chloroform layer (bottom layer) by centrifuging at 25°C, 1,000 × g for 10 min. To the leftover aqueous layer, 1 mL of chloroform was added before repeating the same procedure. The separated chloroform layers were combined, and the solvent was distilled off under a nitrogen stream. After adding 100 *μ*L of the HPLC mobile phase to the residue to dissolve it, the solution was centrifuged at 25°C, 10,000 × g for 5 min, and the supernatant was used as a sample solution.  Table 3 shows the HPLC (Shimazu Corporation) measurement conditions. From the recorded chromatogram, the peak area ratio of TRZ to ALP was quantified, and the blood TRZ concentration was calculated. The area under the plasma concentration-time curve (AUC_mean_) was calculated using the average concentration from 4-–6 mice at each time point with the linear trapezoidal rule.

### 2.9. Statistical Analysis

The experimental results were reported as the mean ± standard deviation (SD). The significance testing was performed using Dunnett's test, and statistical significance was set at *p* < 0.05.

## 3. Results

### 3.1. Effect of NYT on Human CYP3A In Vitro

#### 3.1.1. Inhibitory Effect

**Figure 1:**
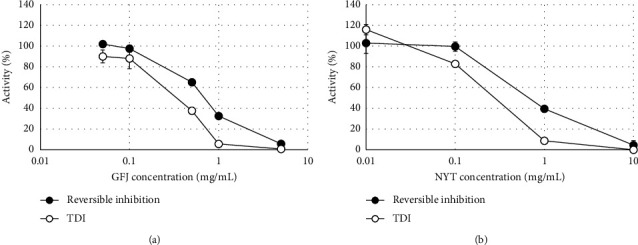
Inhibitory effect of (a) GFJ and (b) NYT on CYP3A. Data are expressed as the mean ± S.D. (*n* = 3). GFJ, grapefruit juice; NYT, Ninjin'yoeito; TDI, time-dependent inhibition.

CYP3A was inhibited in a concentration-dependent manner following the addition of GFJ and NYT. In addition, GFJ and NYT increased the inhibitory effect in the TDI assessment study following 30 min of preincubation (Figures 1(a) and 1(b)). In terms of IC_50_, the reversible inhibition of NYT (0.805) was lower than that of GFJ (0.693). In contrast, for TDI, the IC_50_ of NYT (0.252) was lower than that of GFJ (0.329; Table 4). The IC_50_ shifts (IC_50_ for reversible inhibition/IC_50_ for TDI) for GFJ and NYT after 30 min preincubation were increased 2.11- and 3.19-fold, respectively.

#### 3.1.2. Inductive Effect

The CYP3A4 gene expression increased in a dose-dependent manner from 0.01 mg/mL to 0.1 mg/mL by adding NYT to 0.1% DMSO; however, at 1 mg/mL, the value was similar to that of 0.1% DMSO (Table 5; data are expressed as the mean ± S.D. (*n* = 3)).

### 3.2. Effect of NYT on Mouse CYP3A In Vivo

**Figure 2:**
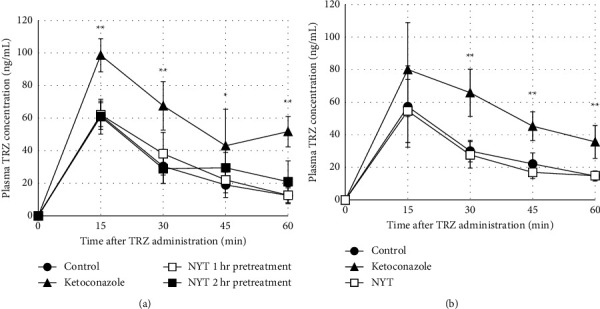
Effect of (a) single administration or (b) repetitive administration for 15 days of NYT on the plasma concentration profiles of TRZ. Data are expressed as the mean ± S.D. (*n* = 4–6). ^*∗*^*p* < 0.05; ^*∗∗*^*p* < 0.01 vs. vehicle group; Dunnett's test. TRZ, triazolam; NYT, Ninjin'yoeito.

In the NYT single-dose test, a significant increase in the blood TRZ concentration was observed in the ketoconazole group compared with the control group at 15, 30, 45, and 60 min after TRZ administration. In contrast, compared with the control group, the NYT 1 h and 2 h pretreatment groups exhibited no significant change in the blood TRZ concentration at all time points (Figure 2(a)). The AUC_mean_ of the NYT 1 h preadministration group (32.2 ng/mL·h) and the NYT 2 h preadministration group (32.5 ng/mL·h) was only 1.096–1.106 times higher than that of the control group (29.3 ng/mL·h), whereas that of the ketoconazole group (58.8 ng/mL·h) was 2.003 times higher. Similarly, in the 15-day NYT repeated-dose test, a significant increase in the blood TRZ concentration was observed in the ketoconazole group compared to the control group at 30, 45, and 60 min after TRZ administration. However, no significant changes were observed in the NYT group relative to the control group at all time points (Figure 2(b)). The AUC_mean_ of the NYT group (26.6 ng/mL·h) was only 0.913 times higher than that of the control group (29.2 ng/mL·h), whereas that of the ketoconazole group (52.2 ng/mL·h) was 1.790 times higher.

## 4. Discussion

NYT, a Japanese formulation of Kampo medicine, is widely used in clinical practice and is effective in patients who are frail with multiple symptoms. The NYT treatment has clinical benefits, including improvements in general health in older adult patients after surgery [4], general malaise and anorexia from cancer treatment [11], coldness and malaise in patients with diabetes [12], and anemia [13, 14]. Furthermore, NYT is often coadministered with western medicine in patients with frailty and who struggle with multiple symptoms. In this study, we investigated the effects of NYT on human liver CYP3A- or mouse liver CYP3A-mediated drug metabolism in vitro and in vivo, respectively.

The results obtained from the in vitro inhibition study using human liver microsomes showed that NYT reversibly inhibited CYP3A in a dose-dependent manner. The inhibitory potency of NYT relative to GFJ was 9.99% when the expected single-dose concentration from each compound was determined by referring to a previous report (data not shown) [15]. This indicates that approximately 10.0 times the amount of administered NYT is required to obtain an inhibitory effect equivalent to that of GFJ, in contrast to a single dose in humans, suggesting that the inhibitory effect of NYT, when taken at the normal dose, is weak. Contrarily, in the TDI assessment, 30 min of preincubation resulted in enhanced CYP3A inhibitory activity of NYT and a 3.19-fold shift in IC_50_ compared to that of the reversible inhibition. The compounds known to have TDI show IC_50_ shifts of ≧ 1.5-fold with 30 min pre-incubation [16]; therefore, NYT is considered to exhibit TDI on CYP3A. In the in vitro induction study using frozen human hepatocytes, compared with 0.1% DMSO administration, NYT administration increased the gene expression of CYP3A4 by more than double. However, compared with the positive control rifampicin, 0.01 and 0.1 mg/mL NYT induced 1.75% and 7.72% CYP3A4 gene expression, respectively. Thus, the CYP3A4 inductive activity of NYT is weaker than that of a known CYP3A4 inducer.

Kampo medicines, which consist of multiple crude drugs, have both enzyme-inhibitory and inductive activity because they contain many components. Of the 12 types of crude drugs that comprise NYT, prior research using human liver microsomes have shown that schisandra, cinnamon bark, licorice, and poria inhibit CYP3A4 [17, 18]. Contrarily, *Schisandra* (gomisin A) and licorice (glycyrrhetinic acid and glycyrrhizic acid) have been reported to induce the expression of CYP3A4 in vitro [19, 20]. The results of the present in vitro study were considered consistent with both inhibitory/inductive activities of the constituent crude drugs reported in these previous studies. However, we considered that the in vitro study alone could not simulate in vivo pharmacokinetics because Kampo medicines are converted into various metabolites in vivo. Therefore, we investigated drug interactions mediated by hepatic CYP3A in vivo using mice.

In the in vivo study, whether a single or repeated doses were administered, NYT had no significant effects on the blood TRZ concentrations in mice relative to the untreated mice. In contrast, ketoconazole, a potent CYP3A4 inhibitor, significantly increased blood TRZ levels in mice. The difference in our results between in vivo and in vitro is also attributed to species differences. However, considering that NYT is converted into various metabolites in vivo, we have determined that this is related not only to species differences but also closely to the differences between in vivo and in vitro. Thus, although crude drugs in NYT contain ingredients that inhibit and induce CYP3A in vitro, we considered that the CYP3A-inhibitory/inductive activities of ingredients in Kampo that have been metabolized in vivo are different from the ones in vitro. Our findings suggest that oral administration of NYT does not affect hepatic CYP3A-mediated drug metabolism in vivo in mice.

Numerous complicated factors cause changes in drug-metabolizing enzyme activities in the human body. In addition to external factors such as the intrusion of foreign substances, drinking, smoking, eating, and taking medicine and various internal factors such as race, sex, age, nutritional status, disease status, and individual differences, genetic factors are also involved [8]. In humans, various combinations of these factors affect the activity of drug-metabolizing enzymes. The hepatic clearance value, which reflects drug metabolism in the liver, is approximately 40% lower in older adults than in adolescents [21]. Among the P450 molecules, CYP3A4 is said to be relatively susceptible to aging [8]. To obtain the full therapeutic effect and reduce drug interactions, the complex involvement of the aforementioned factors should be considered.

## 5. Conclusions

Currently, little is known about the effects of NYT on human CYP3A and mouse CYP3A activity. However, this study provided new evidence for the drug interactions of NYT and suggested that NYT is unlikely to cause liver CYP3A-mediated drug interactions in vivo of mouse. As a result of the recent increase in clinical use of herbal medicines, this report will contribute to understanding the drug interactions between these herbal preparations and other drugs. However, this study does not accurately reflect the pharmacokinetics in humans; thus, these results cannot be directly extrapolated to clinical trials. For the safe and effective use of Kampo medicines containing NYT, further research is necessary regarding pharmacokinetic evidence, including the effect of NYT on the oral clearance of concomitantly administered drugs.

## Figures and Tables

**Table 1:** Herb composition of Kampo formula Ninjin'yoeito (NYT).

Ingredients		Contents (g)
English name	Latin name	
Poria sclerotium	*Poria*	4.0
Japanese angelica root	*Angelicae radix*	4.0
Rehmannia root	*Rehmanniae radix*	4.0
Atractylodes rhizome	*Atractylodis rhizoma*	4.0
Ginseng	*Ginseng radix*	4.0
Cinnamon bark	*Cinnamomi cortex*	3.0
Citrus unshiu peel	*Aurantii nobilis pericarpium*	2.5
Polygala root	*Polygalae radix*	2.0
Peony root	*Paeoniae radix*	2.0
Astragalus root	*Astragali radix*	1.5
Schisandra fruit	*Schisandrae fructus*	1.0
Glycyrrhiza	*Glycyrrhizae radix*	1.0

^*∗*^Approximate 6700 mg of dried water extract of NYT is prepared in a GMP-standardized factory of Kracie Pharma, Ltd. (Japan) based on the above-described composition.

**Table 2:** LC-MS/MS conditions for CYP3A assay HPLC conditions.

Instrument	LC-30AD								
Analytical column	CAPCELL PAK C18 MGIII, 5 *µ*m, 2.0 mm I.D. × 50 mm								
Column oven temperature	40°C (set value)								
Mobile phase	A; 0.1 vol% formic acid								
	B; acetonitrile								
Gradient program	Time (min)	0	1	1.01	2.5	2.51	3.5	3.51	6
	%B	25	25	50	50	90	90	25	25
Flow rate	0.4 mL/min								
Injection volume	10 *µ*L								
Autosampler temperature	4°C (set value)								
Injector rinse solution	Acetonitrile/2-propanol/distilled water (2 : 2 : 1, v/v/v)								
MS/MS condition									
Instrument	QTRAP6500								
Ionization mode	ESI								
Polarity	Positive								
Resolution (Q1/Q3)	Unit/unit								
Duration	6 min								
Scan type	Multiple reaction monitoring								
Detection conditions									
Analyte	Q1 (Da)	Q3 (Da)	Dwell (msec)	DP (V)	EP (V)	CE (V)	CXP (V)		
6*β*-hydroxytestosterone	305.2	269	300	111	10	21	18		
6*β*-hydraoxytestosterone-d_7_	312.21	276.2	300	71	10	21	18		
Curtain gas	40 psi (Nitrogen)								
Ion source gas 1	80 psi (air)								
Ion source gas 2	80 psi (air)								
Spray voltage	5000 V								
Heater temperature	600°C (set value)								
Collision gas	Level 12								

**Table 3:** HPLC conditions for plasma TRZ analysis.

Instrument	Nexera-i LC-2040C 3D
Analytical column	Inertsil ODS-3 (4.6 mm I.D. × 250 mm, 5 *μ*m, GL sciences)
Guard column	Inertsil ODS-3 (4.6 mm I.D. × 33 mm, 5 *μ*m, GL sciences)
Column oven temperature	40°C
Mobile phase	Distilled water/acetonitrile/methanol (60 : 38 : 2, v/v/v)
Flow rate	1.0 mL/min
Injection volume	20 *µ*L
Detector	PDA (wavelength: 222 nm)

**Table 4:** The half-maximum inhibitory concentration (IC_50_) of GFJ and NYT on CYP3A.

Samples	IC_50_ (mg/mL)	
	Reversible inhibition	TDI
GFJ	0.693	0.329
NYT	0.805	0.252

GFJ, grapefruit juice; NYT, Ninjin'yoeito; TDI, time-dependent inhibition.

**Table 5:** Effect of NYT on CYP3A4 mRNA expression.

Samples	mRNA relative expression (CYP3A4/GAPDH)	% (vs. rifampicin)
0.1% DMSO	1.00 ± 0.10	—

20 *μ*mol/L (16.5 mg/L) rifampicin	135 ± 11	—

0.01 mg/L NYT	2.54 ± 0.11	1.75
0.1 mg/L NYT	11.2 ± 0.2	7.72
1 mg/L NYT	1.06 ± 0.05	127.4

NYT, Ninjin'yoeito.

## Data Availability

The data are available on reasonable request from the corresponding author.

## Supplementary Materials

Supplementary Materials 1. HPLC analysis of blood TRZ concentrations: the analytical method validation with all parameters evaluated (such as, peak confirmation, lower limit of quantification, selection of internal standard, calibration curves, selectivity, spike, and recovery).
