# Supplementary material for: Effects of Ninjin'yoeito on Human CYP3A and Mouse CYP3A Activity
Source: Evid Based Complement Alternat Med. 2023 Aug 2;2023:8657478. doi: 10.1155/2023/8657478 (PMC10412298; doi:10.1155/2023/8657478)
Supplement: Supplementary Materials — 1. HPLC analysis of blood TRZ concentrations: the analytical method validation with all parameters evaluated (such as, peak confirmation, lower limit of quantification, selection of internal standard, calibration curves, selectivity, spike, and recovery). [file 8657478.f1.docx]

**Investigation of triazolam quantification in mouse blood**

Analysis by HPLC

The analytical method was reviewed based on the literature [10] and the "Guidelines for Validation of Analytical Methods for Drug Concentration in Biological Samples in Drug Development" issued by the Ministry of Health, Labour and Welfare of Japan .

- Reagents

Table 1　Reagents used

| Name | Grade | Manufacturer |
| --- | --- | --- |
| triazolam | Biochemistry grade | Wako Pure Chemical Industries, Ltd. |
| alprazolam | Biochemistry grade | Wako Pure Chemical Industries, Ltd. |
| methanol | HPLC grade | FUJIFILM Wako Pure Chemical Corporation |
| acetonitrile | HPLC grade | FUJIFILM Wako Pure Chemical Corporation |
| 2-propanol | Precision analysis grade | FUJIFILM Wako Pure Chemical Corporation |

- HPLC conditions

System: Nexera-i LC-2040C 3D (Shimadzu Corporation)

Column: Inertsil ODS-3 (4.6 mm I.D.×250 mm, 5 μm, GL Sciences)

Guard column: Inertsil ODS-3 (4.6 mm I.D.×33 mm, 5 μm, GL Sciences)

Mobile phase: water/ acetonitrile / methanol (60:38:2)

Column temperature: 40 ℃

Detector: PDA (Main wavelength: 222 nm)

Flow rate: 1.0 mL/min

Injection volume: 20 μL

- Sample preparation

The following is a partial modification based on Reference [2].

A 30 μL of the internal standard solution (IS solution) was added to 330 μL of 2-propanol and then to 300 μL of plasma, and mixed. It was then centrifuged (4 ℃, 10000 × *g*, 5 minutes) after standing still for 10 minutes at 4°C, then the supernatant solution separated. A 450 μL of boric acid buffer (50 mM, pH 11.0) was added to 1 mL of chloroform, and then added to the supernatant solution. This was then shakes, centrifuged (25 ℃, 1000 × *g*, 10 min), and the chloroform layer separated. A 1 mL of chloroform was added to the remaining aqueous layer and manipulated in the same manner. The separated chloroform layers were combined and the solvent distilled away under a nitrogen stream. A 100 μL of HPLC mobile phase was added to the residue, dissolved, centrifuged (25°C, 10,000 *g*, 5 min), and then the supernatant was used as the sample solution.

1. Peak confirmation, lower limit of quantification

Peak retention times and peak shapes were confirmed by analyzing TRZ standard solutions. Absorption spectra were also obtained using a PDA detector, and the measurement wavelengths were investigated at the same time.

[Experiment]

- - Preparation of standard solution

Two milligrams of triazolam was accurately taken and diluted to 20 mL with methanol to make a TRZ standard stock solution (100 μg/mL). A 1,000 ng/mL and 100 ng/mL standard solutions were prepared by diluting the TRZ standard stock solution with HPLC mobile phase.

[Results]

 The analysis of each standard solution confirmed the clear peak of TRZ, and there was no problem with the peak shape (Fig. 1). The absorption spectrum showed that the absorption maximum was near 222 nm, so the detection wavelength was set to 222 nm. The lower limit of quantification was approximately 10 ng/mL and the detection limit was approximately 3 ng/mL, assuming that the concentration with S/N 10 was the lower limit of quantification and that with S/N3 was the detection limit. Considering that the sample is concentrated 3-fold by sample preparation, the lower limit of quantification is equivalent to approximately 3 ng/mL in blood, and the detection limit is approximately 1 ng/mL, confirming that analysis is possible even at low concentrations.

Fig. 1　Results of analysis of TRZ standard

1. Selection of internal standard

In this measurement, since the samples were biological samples, there was a possibility of variations in sample preparation due to differences between individual mice, and a certain amount of loss was assumed to occur during sample preparation such as liquid-liquid distribution, so it was decided to perform quantification using the internal standard method to compensate for these variations.

As an internal standard (IS), a substance that has a retention time close to that of the test component and that is completely separated from both peaks is suitable. For LC-MS or LC-MS/MS, a stable isotope-labelled test component is most suitable because separation by mass is possible. However, since the measurement is performed with a UV detector, compounds with identical retention times cannot be used. In addition to the above, since IS was added in this study to compensate for losses during sample preparation, compounds with similar recovery rates as the test component were selected as candidates.

Alprazolam (ALP) was selected as a candidate compound that meets the above conditions because it is readily available and relatively inexpensive (Fig. 2). ALP has the same backbone as TRZ, but with one -Cl removed from TRZ, so it is expected to have similar chemical properties.

In this study, in order to examine the appropriateness of ALP as an IS in the analysis of TRZ, it was decided to confirm that the retention times of TRZ and ALP were as close as possible and that they were completely separated. In subsequent studies, the equivalence of the TRZ and ALP spike and recovery rates was examined in "5. Spike and recovery".


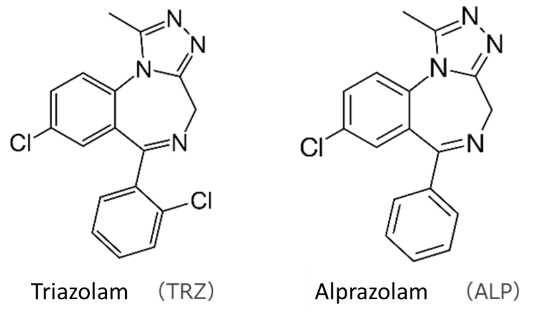


Fig. 2　TRZ and ALP structures

[Experiment]

- - Preparation of mixed standard solutions

Mixed solution of TRZ and ALP: TRZ and ALP were prepared in mobile phase to a final concentration of 1,500 ng/mL and 300 ng/mL, respectively.

[Results]


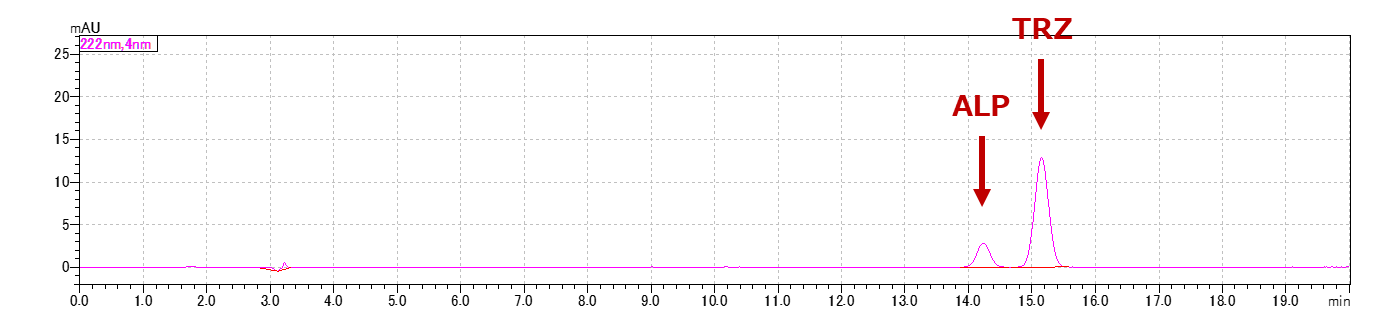
　 ALP had a retention time close to that of TRZ and was found to be completely separated with a resolution of 2.308 (Fig. 3).

Fig. 3　Results of analysis of ALP and TRZ

1. Calibration curves

[Experiment]

Calibration curves were generated for seven concentrations (5, 10, 20, 50, 100, 200, and 500 ng/mL), including 5 ng/mL in blood, which was near the lower limit of quantification, and 500 ng/mL in blood, which was above the maximum concentration expected. In the case of this sample preparation method, the concentration in the sample solution was three times the blood concentration, so the TRZ concentrations were prepared to be 15, 30, 60, 150, 300, 600, and 1,500 ng/mL (including 300 ng/mL of ALP as an internal standard).

[Results]

　The calibration curve showed good linearity with y=0.9346x-0.0100 and R2=0.99996 (Fig. 4). The true values of each concentration of standard samples, within the calibration curve, was obtained from the regression equation; and showed a good agreement with the theoretical value, with a difference of less than 10% for all concentrations (Table 2).


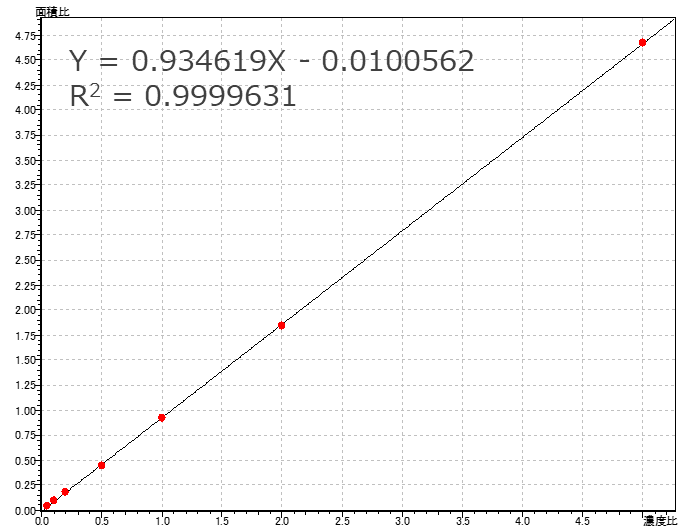
Fig. 4　Calibration curve is generated.

Table 2　 Difference between theoretical and measured values at each calibration point

| Theoretical concentration | Quantitative value | Quantitative value relative to theoretical value (%) |
| --- | --- | --- |
| 4 | 4.4 | 110.0 |
| 20 | 20.2 | 101.0 |
| 40 | 40.4 | 101.0 |
| 100 | 99.6 | 99.6 |
| 200 | 198.5 | 99.3 |
| 400 | 400.8 | 100.2 |

1. Selectivity

　It was confirmed that no peaks originating from interfering substances were observed in the manipulated blank and blank samples.

[Experiment]

No peaks that would interfere with the measurement were detected in the blank plasma.

[Results]

Analysis of blank plasma (matrix sample pretreated without addition of analytes) using a glass instrument confirmed that there were no problems with selectivity, as no impurity peaks overlapping with ALP and TRZ were observed (Fig. 5).


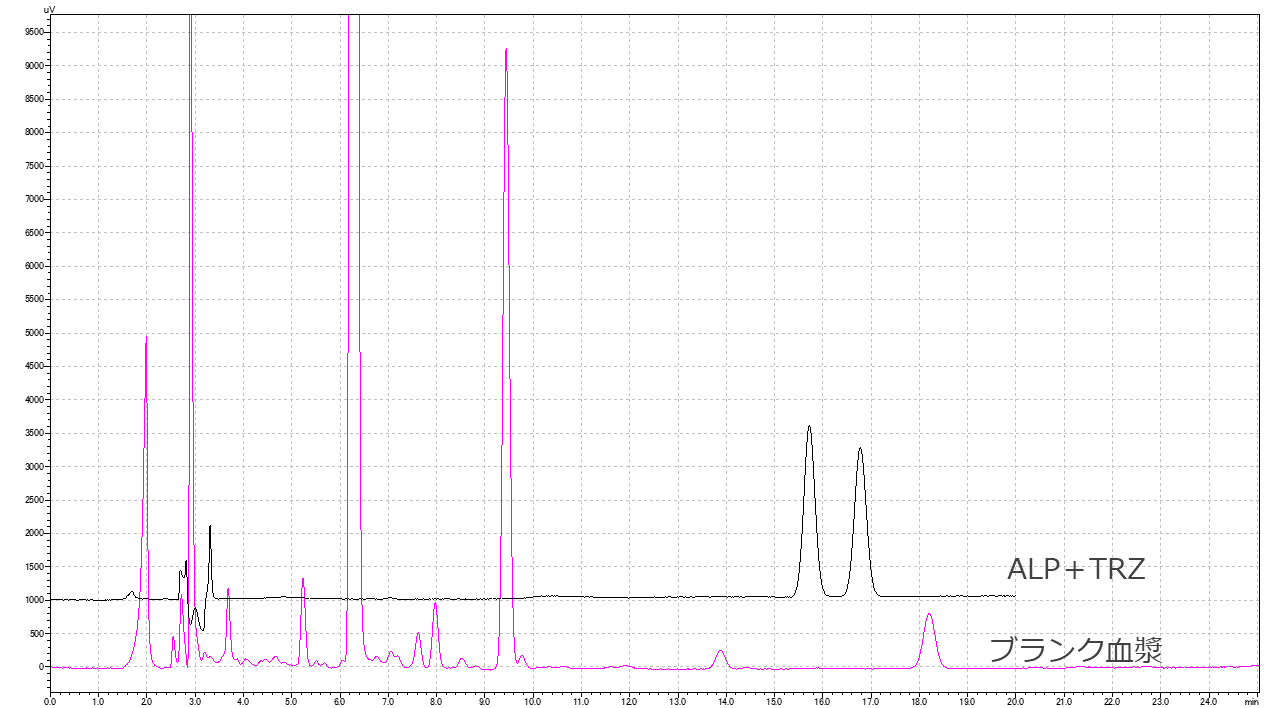


Blank　Plasma

Fig. 5　Results of measurement of blank plasma

1. Spike and recovery

　To evaluate the validity of using ALP as IS, the spike and recovery rate of TRZ and ALP was checked. First, a known amount of a mixed solution of ALP and TRZ was added using water instead of plasma, followed by the preparation and analysis of the resulting sample solution. The blank plasma was then used for the spike and recovery test. In this study, two concentrations, low (equivalent to 10 ng/mL in blood) and high (equivalent to 200 ng/mL in blood), were prepared at n=3 each.

[Experiment]

- Preparation of standard solution for spiking

TRZ and ALP were dissolved in a small amount of 2-propanol, and then prepared in water to make a mixed solution of 2,000 ng/mL TRZ and 1,000 ng/mL ALP for high concentration and 100 ng/mL TRZ and 1,000 ng/mL ALP for low concentration.

- Spike and recovery test (water)

A sample preparation was created after adding 30 μL of the above standard solution instead of the IS solution to 300 μL of water. The obtained sample solution was analyzed and the recovery was calculated by the absolute calibration curve method.

- Spike and recovery test (plasma)

A sample preparation was produced after adding 30 μL of the above standard solution instead of IS solution to 300 μL of blank plasma. The obtained sample solution was analyzed and the recovery rate was calculated by the internal standard method.

[Results]

　Table 3 shows the results of the spike and recovery test conducted using water instead of plasma.

　The good recovery rates of 91-98% were obtained with low spike concentration of TRZ, high spike concentration of TRZ, and ALP. As there was almost no difference between the recovery rates of TRZ and ALP, we thought that variability during sample preparation could be compensated by using ALP for IS.

Table 3　Results of spike and recovery test (water)

| Compound | Spike concentration | Spike and recovery test |
| --- | --- | --- |
| TRZ | 10 ng/mL | 91.6% |
| TRZ | 200 ng/mL | 97.8% |
| ALP | 100 ng/mL | 97.6% |

Table 4 shows the results of the spike and recovery test performed using blank plasma. At high concentrations, both recovery rate and repeatability were good. At low concentrations, the recovery rate was relatively good, although there was some variation.

　One of the reasons for the variation at low concentrations may be the influence of impurities in the plasma samples. Since this test method used chloroform for extraction, impurities may remain on the column during elution with water/acetonitrile/methanol (60:38:2) for 60 minutes, which may have affected the quantitative values because they interfered with the measurement of the next sample. To eliminate this influence, a water/acetonitrile/methanol (10:88:2) flow for about 20 minutes after ALP and TRZ had eluted was used.

Table 4 Results of spike and recovery test (plasma)

| Spike and recovery rate (%) | 1 | 2 | 3 | Mean | RSD (%) |
| --- | --- | --- | --- | --- | --- |
| Low concentration (TRZ 10 ng/mL) | 138.6 | 108.2 | -* | 123.4 | 17.43 |
| High concentration (TRZ 200 ng/mL) | 99.0 | 100.9 | 97.2 | 99.0 | 1.86 |

*：Measurements could not be taken due to damage to the instrument.
